# Fetal Growth Restriction and Clinical Parameters of Newborns from HIV-Infected Romanian Women

**DOI:** 10.3390/medicina59010111

**Published:** 2023-01-05

**Authors:** Simona Claudia Cambrea, Elena Dumea, Lucian Cristian Petcu, Cristina Maria Mihai, Constantin Ghita, Loredana Pazara, Diana Badiu, Costin Ionescu, Mara Andreea Cambrea, Eosefina Gina Botnariu, Florentina Dumitrescu

**Affiliations:** 1 Faculty of Medicine, “Ovidius” University from Constanta, 900470 Constanta, Romania; 2 Clinical Hospital of Infectious Diseases Constanta, 900709 Constanta, Romania; 3 Faculty of Dentistry, “Ovidius” University from Constanta, 900470 Constanta, Romania; 4 “Dr. Alexandru Gafencu” Emergency Military Hospital, Constanta, 900527 Constanta, Romania; 5 Medical Department II, University of Medicine and Pharmacy, “Gr. T. Popa” Iasi, 700115 Iasi, Romania; 6 Department of Infectious Disease, Faculty of Medicine, University of Medicine and Pharmacy of Craiova, 200349 Craiova, Romania

**Keywords:** HIV-positive newborns, anthropometrical parameters, fetal growth restriction, Apgar score, fetal length, birth-related outcomes

## Abstract

*Background and Objectives:* The present study assessed the fetal growth restriction and clinical parameters of both human immunodeficiency virus (HIV)-negative and HIV-positive newborns from HIV-infected mothers in two HIV-acquired immunodeficiency syndrome regional centers (RCs) in Constanta and Craiova, Romania, in order to evaluate the adverse birth-related outcomes. *Materials and Methods:* These represent a retrospective study conducted between 2008 and 2019, in which 408 pregnant HIV-positive women, 244 from Constanta RC and 164 from Craiova RC, were eligible to participate in the study. Consecutive singleton pregnancies delivered beyond 24 weeks of pregnancy were included. Growth restriction in newborns was defined as the birth weight (BW) being less than the third percentile, or three out of the following: BW < 10th percentile; head circumference (HC) < 10th percentile; birth length (BL) < 10th percentile; prenatal diagnosis of fetal growth restriction; and maternal pregnancy information. Of the 244 newborns delivered in Constanta, RC, 17 were HIV-positive, while in Craiova, RC, of the 164 newborns, 9 were HIV-positive. All HIV-positive women were on combined antiretroviral therapy (cART) during pregnancy, similar to all HIV-positive newborns who received ARTs for the first six weeks. We search for the influence of anthropometrical parameters (i.e., HC, BL, and BW), as well as clinical parameters (i.e., newborn sex and Apgar score) for both HIV-negative and HIV-positive newborns, along with the survival rate of HIV-positive newborns. *Results:* There were no differences in the sex of the newborns within either group, with more than 50% being boys. Similarly, the Apgar score did not show any statistically significant values between the two groups (i.e., *p* = 0.544 for HIV-positive newborns vs. *p* = 0.108 for HIV-negative newborns). Interestingly, our results showed that in Craiova, RC, there was a chance of 2.16 to find an HIV-negative newborn with an HC < 10th percentile and a 2.54 chance to find an HIV-negative newborn with a BL < 10th percentile compared to Constanta, RC, without any significant differences. On the contrary, Constanta, RC, represented a higher risk of death (i.e., 3.049 times, *p* = 0.0470) for HIV-positive newborns compared to Craiova, RC. *Conclusions:* Our results support the idea that follow-up of fetal growth restriction should be part of postnatal care in this high-risk population to improve adverse birth-related outcomes.

## 1. Introduction

Starting in 1990, Romania showed an important cohort of the human immunodeficiency virus (HIV)-infected newborns and children, through unsafe parenteral treatments with reusable unsterilized syringes or blood products [1].

In 2021, approximately 38.4 million people worldwide were living with HIV-infection. In Romania, the number of people undergoing combined antiretroviral therapy (cART) and prophylaxis in September 2022 was 13,985, of which 559 new cases of women were detected in the same year [2]. By using diagnostic tools and proper treatment, the prevention of mother-to-child transmission (MTCT) of HIV-infection can be remarkable.

Nowadays, investigating the fetal growth prospective has increased the attention in detecting different newborns’ abnormalities. Understanding the trajectory of fetal growth has become important to prevent birth-related outcomes. It was found that HIV-infection affects more women in comparison to the rest of the population, who tend to seek medical attention at the later stages of the disease as compared to men [3], even under the cART prescription [4]. However, it becomes fundamental to know the effects of HIV-infection on pregnancy or offspring anthropometry, with MTCT representing the main consequences, which also increase the risk of adverse birth-related measures. However, attention must be directed to the potential risk for fetal growth restriction along with other important clinical parameters [5]. Although the relationship between intrauterine exposure of fetuses to HIV-infection and the use of cART remains controversial, some studies have suggested cART as a main risk factor. Therefore, if growth restriction results from a direct effect of the virus, immunosuppression, or associated co-morbidities, it is still under debate [6,7]. The study of Beune and contributors gives the final consensus definition of growth restriction in newborns: birth weight (BW) less than the third percentile, or three out of the following: BW < 10th percentile; head circumference (HC) < 10th percentile; birth length (BL) < 10th percentile; prenatal diagnosis of fetal growth restriction; and maternal pregnancy information [8].

Keeping in mind the social or behavioral risk features related to HIV-infection, growth restriction in newborns and clinical parameters may be shared by the same women, resulting in a high prevalence of adverse newborn outcomes [9].

In the present study, we assessed the anthropometric parameters and clinical features of both HIV-negative and HIV-positive newborns along with the survival rate of HIV-positive newborns from HIV-infected mothers from two HIV-acquired immunodeficiency syndrome (AIDS) Regional Centers (RCs) Constanta and Craiova from Romania in order to evaluate the adverse birth-related outcomes.

## 2. Materials and Methods

We conducted a retrospective study on newborns from HIV-positive mothers in two HIV-AIDS RCs from Romania in terms of monitoring HIV-AIDS infection to assess the incidence of fetal growth restriction and clinical parameters among newborns.

During the study period, between 2008 and 2019, 408 HIV-positive pregnant women were monitored, of whom 244 mothers were from HIV-AIDS Constanta, RC, and 164 mothers were from HIV-AIDS Craiova, RC. The population study consisted of participants > 18 years, with a singleton pregnancy. Exclusion criteria included pre-existing hypertension, diabetes, renal, autoimmune, active opportunistic infection for HIV-positive women, morbid obesity, or drug use.

All HIV-positive women were on cARTs (p.o. administration) during pregnancy and childbirth, being administered cARTs, which consisted of triple therapy based on a combination of nucleotide reverse-transcriptase inhibitors, associated with non-nucleoside reverse-transcriptase inhibitors, protease inhibitors, or integrase inhibitors. The mothers’ HIV infection was established using the national protocol (e.g., 2 serological enzyme-linked immunosorbent assay (ELISA) positive tests, followed by a Western Blot and HIV-ribonucleic acid (RNA)). According to the prevention of MTCT, for all known HIV-positive women it was a planned Caesarian section (C-section) delivery, but emergency C-section delivery was recommended in case of pregnancy complications or newly diagnosed HIV pregnant women’s status who give birth in the hospital. We included pregnant women known as HIV-infected and those who tested HIV-positive before or during labor.

We studied infants from both centers for 18 months, to establish their HIV status, receiving the standard of care from a dedicated physician according to the Romanian National HIV Management Guidelines. The infant’s final HIV status was established after a period of 18 months of follow-up using international recommendations with molecular and serological tests. HIV-RNA determination was performed using COBAS Amplicor TaqMan version 2-0, Roche Molecular Systems Bucharest, Romania (detection limit: 20 copies/mL), and serological tests: ELISA and Western Blot. Newborns received antiretroviral prophylaxis for six weeks. The cARTs used in newborns were zidovudine and lamivudine with/without nevirapine or lopinavir/ritonavir. If, at any point in the follow-up period, the newborn’s HIV viral load was detectable, we considered an HIV-positive newborn. If during the follow-up period all serological tests remain negative and HIV viral load is undetectable, the newborn was considered HIV-negative [10].

We search the anthropometrical parameters (i.e., HC, BL, and BW) for both HIV-negative and HIV-positive newborns, as well as the clinical parameters (i.e., newborn sex and Agar score), along with the survival rate of HIV-positive newborns. We documented the Apgar score by evaluating five parameters: the color, the heart rate, reflexes, muscle tone, and respiration [11]. Neonatal dimensions were measured within 24 h of birth by neonatal doctors or nurses [12]. In fetal death cases, the autopsy dimensions were used.

Consecutive singleton pregnancies delivered beyond 24 weeks of pregnancy were included and confirmed by an ultrasound examination. Growth restriction in newborns was defined as BW < 10th percentile [8].

The statistical analysis was performed using IBM SPSS statistics software version 23. (Armonk, NY, USA: IBM Corp). The procedures used were descriptive statistics, graphs, and non-parametric statistical tests. Data are presented as medians and IQRs (interquartile range) for continuous variables in cases of skewed distributions or as percentages for categorical variables. For hypotheses testing: Independent Samples Mann Whitney U test, Independent Samples Median test, Chi-Square Test of association, and Chi-squared test for the comparison of two proportions were used depending on the type of analyzed variables. Kaplan-Meier survival analysis was performed using MedCalc statistics software version 14.8.1. (MedCalc Software bvba, Ostend, Belgium, 2014). The significance level (α) was set at 0.05.

Informed consent was obtained from all participants in the study as well as the Agreement of the Ethics Commission (No. 33/22 June 2022) for the publication of the data.

## 3. Results

### 3.1. HIV Transmissibility Rates to Newborns

During this period, 244 newborns from Constanta, RC, and 164 newborns from Craiova, RCs, were monitored.

**Figure 1:**
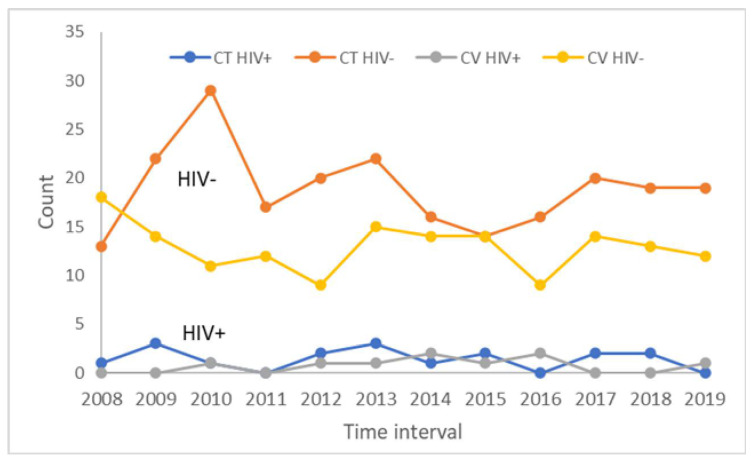
Line chart showing comparative trends over time for HIV-negative and HIV-positive newborns in Constanta and Craiova, Regional Centers (RCs). CT HIV+ = HIV-positive newborns from Constanta RC; CT HIV− = HIV-negative newborns from Constanta RC; CV HIV+ = HIV-positive newborns from Craiova RC; CV HIV− = HIV-negative newborns from Craiova RC.

Of the 244 newborns studied in Constanta, RC, 227 were HIV-negative and 17 were HIV-positive, which represented a transmissibility rate of 6.97%, while in Craiova, RC, among 164 newborns from HIV-positive women, 155 were HIV-negative and 9 were HIV-positive, which represented a transmissibility rate of 5.4% (Figure 1). There was no statistically significant difference between the mentioned proportions (Chi-squared = 0.155, df = 1, *p* = 0.6941).

### 3.2. Clinical and Anthropometrical Parameters of HIV-Positive Newborns

From 26 HIV-positive newborns, 14 (53.84%) were boys (8 from Constanta RC and 6 from Craiova RC) and 12 (46.15%) were girls (9 from Constanta RC and 3 from Craiova RC), with a similar rate in both RCs, without any statistical significance (*p* = 0.340).

The Apgar score in the case of HIV-positive newborns performed at birth was between 6 and 10, with a median value of 8 (IQR = 1) for Constanta RC and between 7 and 10, with a median value of 8 (IQR = 1) for Craiova RC, but without any statistical significance between the two groups (*p* = 0.544, Table 1).

Table 1 presents the anthropological parameters and the Apgar score for HIV-positive newborns from the two RCs.

The test does not reveal significant differences between the HC HIV-positive newborns from Constanta RC (Median = 32, *n* = 17) and the HC HIV-positive newborns from Craiova, RC (Median = 33, *n* = 9), *U*= 62.00, z = −0.789, *p* = 0.430.

**Figure 2:**
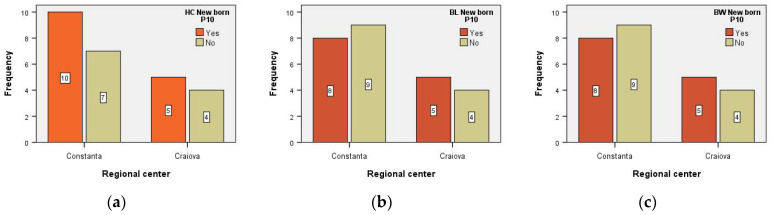
Anthropometric parameters (**a**) head circumference (HC), (**b**) birth length (BL), and (**c**) birth weight (BW) of HIV-positive newborns from the two Regional Centers. P10 = percentile 10.

There was no association between the variables in each RC and the newborns’ HC < 10th percentile (i.e., χ^2^_stat_ = 0.026, df = 1, *p* = 0.873 > α = 0.05). The chance of finding a newborn with HC < 10th percentile present in the group of patients from Constanta RC is equal to the chance of finding a newborn with HC < 10th percentile present in the group of patients from Craiova, RC: OR = 1.143; 95% CI for OR = (0.224, 5.841) (Figure 2).

The test does not reveal significant differences between the BL HIV-positive newborns from Constanta, RC (Median = 48, *n* = 17), and the BL HIV-positive newborns from Craiova, RC (Median = 48, *n* = 9), *U* = 71.50, z = −0.272, *p* = 0.786.

There was no association between the variables in each RC and BL HIV-positive newborns with < 10th percentile (i.e., χ^2^_stat_ = 0.170, df = 1, *p* = 0.680 > α = 0.05). The chance of finding a newborn with BL < 10th percentile present in the group of patients from Constanta, RC, is equal to the chance of finding a newborn with BL < 10th percentile present in the group of patients from Craiova, RC: OR = 0.711; 95% CI for OR = (0.140, 3.606) (Figure 2).

The test does not reveal significant differences between the BW HIV-positive newborns from Constanta, RC (Median = 2800.00, *n* = 17), and the BW HIV-positive newborns from Craiova, RC (Median = 2640.00, *n* = 9), *U* = 74.50, z = −0.108, *p* = 0.914.

There was no association between the variables of each RC and the BW HIV-positive newborns (i.e., χ^2^_stat_ = 0.170, df = 1, *p* = 0.680 > α = 0.05). The chance of finding a newborn with BW < 10th percentile present in the group of patients from Constanta, RC, is equal to the chance of finding a patient with BW < 10th percentile present in the group of patients from Craiova, RC: OR = 0.711; 95% CI for OR = (0.140, 3.606) (Figure 2).

### 3.3. Clinical and Anthropometrical Parameters of HIV-Negative Newborns

From 382 HIV-negative newborns, 194 (50.78%) were boys (121 from Constanta, RC, and 73 from Craiova, RC), and 188 (49.21%) were girls (106 from Constanta, RC, and 82 from Craiova, RC), with a similar rate in both RCs but without any statistical significance (*p* = 0.233).

The Apgar score in the case of HIV-negative newborns performed at birth, was between 4 and 10, with a median value of 9 (IQR = 1) for Constanta, RC, and between 5 and 9, with a median value of 9 (IQR = 1) for Craiova, RC, without any statistical significance between the two groups (*p* = 0.108, Table 2).

Table 2 presents the anthropological parameters and the Apgar score for HIV-negative newborns from the two RCs.

The test does not reveal significant differences between the HC HIV-negative newborns from Constanta, RC (Median = 33, *n* = 227), and the HC HIV-negative newborns from Craiova, RC (Median = 33, *n* = 155), *U* = 16693.5, z = −0.861, *p* = 0.389.

**Figure 3:**
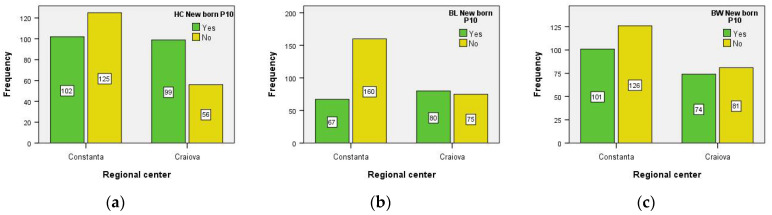
Anthropometric parameters (**a**) head circumference (HC), (**b**) birth length (BL), and (**c**) birth weight (BW) of HIV-negative newborns from the two Reginal Centers. P10 = percentile 10.

There was an association between the variables in each RC and the HC HIV-negative newborns (i.e., χ^2^_stat_ = 13.249, df = 1, *p* < 0.001 < α = 0.05). The chance of finding a newborn with the HC < 10th percentile present in the group of patients from Constanta, RC, is 2.16 (1/0.462) times lower than the chance of finding a patient with the HC < 10th percentile present in the group of patients from Craiova, RC: OR = 0.462; 95% CI for OR = (0.303, 0.702) (Figure 3).

The test does not reveal significant differences between the BL HIV-negative newborns from Constanta, RC (Median = 48.00, *n* = 227), and BL HIV-negative newborns from Craiova, RC (Median = 48, *n* = 155), *U* = 17269, z = −0.308, *p* = 0.758.

There was an association between the variables in each RC and the BL HIV-negative newborns (i.e., χ^2^_stat_ = 18.999, df = 1, *p* < 0.001 < α = 0.05). The chance of finding a newborn with BL < 10th percentile present in the group of patients from Constanta, RC, is 2.54 (1/0.393) times lower than the chance of finding a newborn with BL < 10th percentile present in the group of patients from Craiova, RC: OR = 0.393; 95% CI for OR = (0.257, 0.600) (Figure 3).

The test does not reveal significant differences between the BW HIV-negative newborns from Constanta, RC (Median = 2800.00, *n* = 227), and the BW HIV-negative newborns from Craiova, RC (Median = 2830, *n* = 155), *U* = 16296.5, z = −1.224, *p* = 0.221.

There was no association between the variables in each RC and the BW HIV-negative newborns (i.e., χ^2^_stat_ = 0.392, df = 1, *p* = 0.531 > α = 0.05). The chance of finding a newborn with BW < 10th percentile present in the group of patients from Constanta, RC, is equal to the chance of finding a newborn with BW < 10th percentile present in the group of patients from Craiova, RC: OR = 0.877; 95% CI for OR = (0.582, 1.322) (Figure 3).

### 3.4. Survival of HIV-Positive Newborns

**Figure 4:**
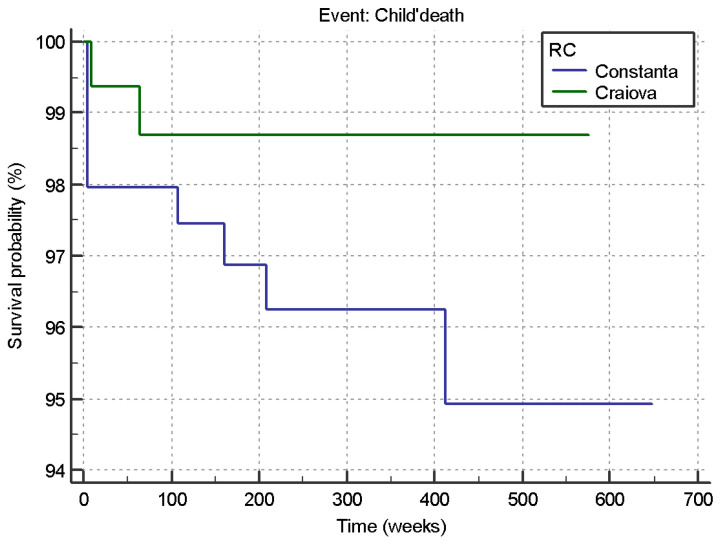
Survival curve of HIV-positive newborns for each RC.

From the 408 newborns from the HIV-positive women analyzed in the two RCs, 9 infants (3.69%) from the 17 HIV-positive from Constanta, RC, and 2 infants (1.22%) from the 9 HIV-positive from Craiova, RC, reached the final event, death (Figure 4).

The two survival curves differ significantly: Chi-square = 3.945, df = 1, *p* = 0.0470 < 0.05. Therefore, the estimated risk of death in the case of newborns from Constanta, RC, was 3.049 times higher than that in Craiova, RC (the risk ratio HR = 3.042 and the 95% CI for HR = (1.0844 to 10.3381)).

## 4. Discussion

The staff at our clinic continues to improve the healthcare provided to HIV-infected patients, especially the women of childbearing age, to allow them the chance to have a healthy child [13,14,15,16,17,18]. Our study shows that there were no differences in the sex of the newborns between the Constanta and Craiova RCs, with more than 50% being boys. Similarly, the Apgar score didn’t show any statistically significant values between the two groups (i.e., *p* = 0.544 for HIV-positive infants vs. *p* = 0.108 for HIV-negative infants).

There was no dependent relationship between the variables in each RC and fetal growth restriction (i.e., χ^2^_stat_ = 0.454, df = 1, *p* = 0.500 > α = 0.05) in the HIV-positive newborns. The chance of finding a newborn with growth restriction present in the group of patients from Constanta, RC, was equal to the chance of finding a newborn with growth restriction present in the group of patients from Craiova, RC: OR = 1778; 95% CI for OR = (0.331, 9.554).

There was no dependent relationship between the variables in each RC and fetal growth restriction (i.e., χ^2^_stat_ = 2.213, df = 1, *p* = 0.137 > α = 0.05) in the HIV-negative newborns. The chance of finding a newborn with growth restriction present in the group of patients from Constanta, RC, was equal to the chance of finding a newborn with growth restriction present in the group of patients from Craiova, RC: OR = 0.704; 95% CI for OR (0.443, 1.119).

Therefore, in Craiova, RC, there was a 2.16 percent chance of finding a newborn with an HC < 10th percentile compared to Constanta, RC. Similarly, in the same region, there was also a 2.54 chance of finding a newborn with a BL < 10th percentile compared to the Constanta, RC, being at higher risk of fetal growth restriction.

However, the Constanta RC represented a higher risk of death (i.e., 3.049 times, *p* = 0.0470) among HIV-positive newborns compared to the Craiova RC.

If, in the case of anthropometric parameters (i.e., HC and BL), Craiova RC presented a higher risk compared to Constanta RC, and in the case of the estimated risk of death, Constanta RC presented a higher risk compared to Craiova RC. The effect of HIV-infection risk factors on adverse perinatal outcomes has not yet been disclosed. Risk factors such as poverty, lack of social support, anemia, diabetes, hypertension, chemo- or radiotherapy including different oncologic treatments [19], bariatric surgery involvement [20], or other pathogenic agents similar to hyper-virulent *Klebsiella pneumoniae* [21] could have important involvement on growth restriction, preterm birth, or even mortality of newborns [22].

The most dangerous consequences for newborns occur when the mother is primarily infected in the first trimester of pregnancy. Although a vaccine is not actually available to avoid HIV-primary infection, prevention through hygienic measures represents the only way to prevent infections. These mainly include avoiding contact with children and washing hands thoroughly after this kind of contact [23].

Therefore, it is difficult to notice the independent feature of each risk factor in women with HIV-infection and to be able further to make clear preventions [24].

However, some studies showed low BW or premature children born from HIV-infected mothers [25,26,27]. We did not, however, find a significantly increased risk of low BW in women with HIV-infection in the present study.

Delicio and contributors showed high rates of neonatal adverse outcomes on 793 pregnancies from HIV-positive mothers. About 22.5% of the children had low BW, 22% were born prematurely, 18% were SGA, and 4% had very low BW [28]. Another study had similar results, with 74 children exposed to maternal cART between 2001 and 2012, in which 34.8% were exposed since conception, preterm birth rates of 17.5%, and low BW of 20.2%, with higher rates of HIV-infected mothers [9].

In the case of cART exposure, it showed a higher incidence of prematurity or low BW in children. Moreover, C-section was linked with a higher occurrence in low BW, being used especially in chronic distress or low fetal supply. National guidelines from 23 European countries recommended that HIV-positive women on successful cART with a very low or undetectable viral load < 1000 can have a vaginal delivery [29].

In one of our previous studies from the Constanta RC, the mean BW was 2670 g, and the percentage of BW < 10th percentile was 58.05%. Infants who presented < 10th percentile for weight and length were 22.76%. About 11.38% of infants were < 10th percentile for weight, length, and cranial circumference [30]. However, our results did not show any association between the two RCs, with different materno-fetal monitoring possibilities and the presence of fetal growth restriction.

## 5. Conclusions

HIV-positive women who received cART did not appear to have newborns with abnormal fetal growth, as evidenced by no significant difference in anthropometrical or clinical parameters between both HIV-positive and HIV-negative newborns. Finally, our results support the notion that follow-up of fetal growth restriction should be part of postnatal care in this high-risk population to improve adverse birth-related outcomes.

## Figures and Tables

**Table 1:** Anthropometric parameters and Apgar score of HIV-positive newborns.

Infant Parameters	Regional Center	Minim	Maxim	Median	IQR	*p*
HC (cm)	Constanta	23.00	35.00	32.00	5.00	0.430
	Craiova	26.00	35.00	33.00	4.25	
BL (cm)	Constanta	38.00	51.00	48.00	5.00	0.786
	Craiova	37.00	50.00	48.00	3.50	
BW (g)	Constanta	1500.00	3500.00	2800.00	975.00	0.914
	Craiova	1100.00	3900.00	2640.00	1085.00	
Apgar Score	Constanta	6.00	10.00	8.00	1.00	0.544
	Craiova	7.00	10.00	8.00	1.00	

HC = head circumference; BL = birth length; BW = birth weight; IQR = interquartile range (75th percentile (P75)–25th percentile (P25)).

**Table 2:** Anthropometric parameters and Apgar score of HIV-negative newborns.

Infant Parameters	Regional Center	Minim	Maxim	Median	IQR	*p*
HC (cm)	Constanta	23.00	38.00	33.00	3.00	0.389
	Craiova	24.00	37.00	33.00	2.00	
BL (cm)	Constanta	35.00	55.00	48.00	4.00	0.758
	Craiova	35.00	52.00	48.00	3.00	
BW (g)	Constanta	1000.00	4000.00	2800.00	700.00	0.221
	Craiova	950.00	4300.00	2830.00	720.00	
Apgar Score	Constanta	4.00	10.00	9.00	1.00	0.108
	Craiova	5.00	9.00	9.00	1.00	

HC = head circumference; BL = birth length; BW = birth weight, IQR = interquartile range (75th percentile (P75)–25th percentile (P25)).

## Data Availability

The data of this report are available from the corresponding authors upon request.
